# Supplementary material for: Unveiling the Bioactive Potential of the Invasive Jellyfish Phyllorhiza punctata Through Integrative Transcriptomic and Proteomic Analyses
Source: Biomolecules. 2025 Aug 4;15(8):1121. doi: 10.3390/biom15081121 (PMC12383608; doi:10.3390/biom15081121)
Supplement: Supplementary file 1 [file biomolecules-15-01121-s001.zip › Suplementary Material/FileS3/PEPFOLD4/XP_065068582_15006/clusters.html]

 PEP-FOLD clusters


Download archive of all models  here   

|  |  |  |
| --- | --- | --- |
| # | file | sOPEP |
| # cluster 1 - Size: 88 |  |  |
|  | XP\_065068582\_15006-model1 | -16.2782 |
|  | XP\_065068582\_15006-model1.1 | -16.2782 |
|  | XP\_065068582\_15006-model1.2 | -16.2782 |
|  | XP\_065068582\_15006-model1.3 | -16.2782 |
|  | XP\_065068582\_15006-model1.4 | -16.2782 |
|  | XP\_065068582\_15006-model1.5 | -16.2782 |
|  | XP\_065068582\_15006-model1.6 | -16.2782 |
|  | XP\_065068582\_15006-model1.7 | -16.2782 |
|  | XP\_065068582\_15006-model1.8 | -16.2782 |
|  | XP\_065068582\_15006-model1.9 | -16.2782 |
|  | XP\_065068582\_15006-model1.10 | -16.2782 |
|  | XP\_065068582\_15006-model1.11 | -16.2782 |
|  | XP\_065068582\_15006-model1.12 | -16.2782 |
|  | XP\_065068582\_15006-model1.13 | -16.2488 |
|  | XP\_065068582\_15006-model1.14 | -16.2488 |
|  | XP\_065068582\_15006-model1.15 | -16.2488 |
|  | XP\_065068582\_15006-model1.16 | -16.2488 |
|  | XP\_065068582\_15006-model1.17 | -16.2488 |
|  | XP\_065068582\_15006-model1.18 | -16.2488 |
|  | XP\_065068582\_15006-model1.19 | -16.2488 |
|  | XP\_065068582\_15006-model1.20 | -16.2391 |
|  | XP\_065068582\_15006-model1.21 | -16.2391 |
|  | XP\_065068582\_15006-model1.22 | -16.2391 |
|  | XP\_065068582\_15006-model1.23 | -16.2391 |
|  | XP\_065068582\_15006-model1.24 | -16.2391 |
|  | XP\_065068582\_15006-model1.25 | -16.2391 |
|  | XP\_065068582\_15006-model1.26 | -16.2391 |
|  | XP\_065068582\_15006-model1.27 | -16.2391 |
|  | XP\_065068582\_15006-model1.28 | -16.2391 |
|  | XP\_065068582\_15006-model1.29 | -16.2391 |
|  | XP\_065068582\_15006-model1.30 | -16.2391 |
|  | XP\_065068582\_15006-model1.31 | -16.2391 |
|  | XP\_065068582\_15006-model1.32 | -16.2391 |
|  | XP\_065068582\_15006-model1.33 | -16.2391 |
|  | XP\_065068582\_15006-model1.34 | -16.2391 |
|  | XP\_065068582\_15006-model1.35 | -16.2391 |
|  | XP\_065068582\_15006-model1.36 | -16.2391 |
|  | XP\_065068582\_15006-model1.37 | -16.2391 |
|  | XP\_065068582\_15006-model1.38 | -16.2391 |
|  | XP\_065068582\_15006-model1.39 | -16.2318 |
|  | XP\_065068582\_15006-model1.40 | -16.2318 |
|  | XP\_065068582\_15006-model1.41 | -16.2318 |
|  | XP\_065068582\_15006-model1.42 | -16.2318 |
|  | XP\_065068582\_15006-model1.43 | -16.2318 |
|  | XP\_065068582\_15006-model1.44 | -16.2318 |
|  | XP\_065068582\_15006-model1.45 | -16.2318 |
|  | XP\_065068582\_15006-model1.46 | -16.2318 |
|  | XP\_065068582\_15006-model1.47 | -16.2318 |
|  | XP\_065068582\_15006-model1.48 | -16.2318 |
|  | XP\_065068582\_15006-model1.49 | -16.2318 |
|  | XP\_065068582\_15006-model1.50 | -16.2318 |
|  | XP\_065068582\_15006-model1.51 | -16.2318 |
|  | XP\_065068582\_15006-model1.52 | -16.2318 |
|  | XP\_065068582\_15006-model1.53 | -16.2318 |
|  | XP\_065068582\_15006-model1.54 | -16.2318 |
|  | XP\_065068582\_15006-model1.55 | -16.2318 |
|  | XP\_065068582\_15006-model1.56 | -16.2318 |
|  | XP\_065068582\_15006-model1.57 | -16.2318 |
|  | XP\_065068582\_15006-model1.58 | -16.2318 |
|  | XP\_065068582\_15006-model1.59 | -16.2318 |
|  | XP\_065068582\_15006-model1.60 | -16.2318 |
|  | XP\_065068582\_15006-model1.61 | -16.2318 |
|  | XP\_065068582\_15006-model1.62 | -16.2318 |
|  | XP\_065068582\_15006-model1.63 | -16.2318 |
|  | XP\_065068582\_15006-model1.64 | -16.2318 |
|  | XP\_065068582\_15006-model1.65 | -16.2318 |
|  | XP\_065068582\_15006-model1.66 | -16.2318 |
|  | XP\_065068582\_15006-model1.67 | -16.2318 |
|  | XP\_065068582\_15006-model1.68 | -16.2318 |
|  | XP\_065068582\_15006-model1.69 | -16.2318 |
|  | XP\_065068582\_15006-model1.70 | -16.2318 |
|  | XP\_065068582\_15006-model1.71 | -16.2318 |
|  | XP\_065068582\_15006-model1.72 | -16.2202 |
|  | XP\_065068582\_15006-model1.73 | -16.2202 |
|  | XP\_065068582\_15006-model1.74 | -16.2202 |
|  | XP\_065068582\_15006-model1.75 | -16.2111 |
|  | XP\_065068582\_15006-model1.76 | -16.2111 |
|  | XP\_065068582\_15006-model1.77 | -16.2111 |
|  | XP\_065068582\_15006-model1.78 | -16.185 |
|  | XP\_065068582\_15006-model1.79 | -16.1387 |
|  | XP\_065068582\_15006-model1.80 | -16.1301 |
|  | XP\_065068582\_15006-model1.81 | -16.0596 |
|  | XP\_065068582\_15006-model1.82 | -16.0596 |
|  | XP\_065068582\_15006-model1.83 | -16.0524 |
|  | XP\_065068582\_15006-model1.84 | -15.8738 |
|  | XP\_065068582\_15006-model1.85 | -15.6941 |
|  | XP\_065068582\_15006-model1.86 | -15.6884 |
|  | XP\_065068582\_15006-model1.87 | -15.5766 |
| # cluster 2 - Size: 2 |  |  |
|  | XP\_065068582\_15006-model2 | -7.99812 |
|  | XP\_065068582\_15006-model2.1 | -6.18168 |
| # cluster 3 - Size: 2 |  |  |
|  | XP\_065068582\_15006-model3 | -7.58698 |
|  | XP\_065068582\_15006-model3.1 | -7.39605 |
| # cluster 4 - Size: 2 |  |  |
|  | XP\_065068582\_15006-model4 | -7.47158 |
|  | XP\_065068582\_15006-model4.1 | -6.67086 |
| # cluster 5 - Size: 1 |  |  |
|  | XP\_065068582\_15006-model5 | -7.16398 |
| # cluster 6 - Size: 1 |  |  |
|  | XP\_065068582\_15006-model6 | -7.14462 |
| # cluster 7 - Size: 2 |  |  |
|  | XP\_065068582\_15006-model7 | -6.21717 |
|  | XP\_065068582\_15006-model7.1 | -6.01241 |
| # cluster 8 - Size: 1 |  |  |
|  | XP\_065068582\_15006-model8 | -6.11148 |
| # cluster 9 - Size: 1 |  |  |
|  | XP\_065068582\_15006-model9 | -5.99163 |
|  |  |  |
